# A Cancer-Favoring, Engineered Vaccinia Virus for Cholangiocarcinoma

**DOI:** 10.3390/cancers11111667

**Published:** 2019-10-27

**Authors:** So Young Yoo, Narayanasamy Badrinath, Hye Lim Lee, Jeong Heo, Dae-Hwan Kang

**Affiliations:** 1 BIO-IT Foundry Technology Institute, Pusan National University, Busan 46241, Korea; 2 Research Institute for Convergence of Biomedical Science and Technology, Pusan National University Yangsan Hospital, Yangsan 50612, Korea; roasua@hanmail.net (H.L.L.); sulsulpul@naver.com (D.-H.K.); 3 Biomedical Sciences, School of Medicine, Pusan National University, Yangsan 50612, Korea; badrisamy@gmail.com (N.B.); sodium@korea.com (J.H.)

**Keywords:** cholangiocarcinoma (CCA), cancer-favoring vaccinia virus (CVV), alkaline extracellular microenvironment

## Abstract

While oncolytic vaccinia virus-based therapy has shown promising results for uncured patients with cancer, its effects on cholangiocarcinoma (CCA) remain unclear. Here, we evaluated the anti-cancer activity of the cancer-favoring oncolytic vaccinia virus (CVV), which was recognized as a promising therapy for stem cell-like colon cancer cells (SCCs) and metastatic hepatocellular carcinoma (HCC) in previous studies. CCA presents major challenges, such as clinical complexity, stem cell cancer characteristics, a high refractory rate, resistance to conventional therapy, and a dismal prognosis. In the present study, we confirmed the oncolytic activity of the CVV in CCA with a slightly alkaline microenvironment (pH 7–8), in which the CVV was stable and highly effective at infecting CCA. Taken together, our findings suggest that CVV-based therapy is highly suitable for the treatment of CCA.

## 1. Introduction

DNA recombination technology provides unprecedented opportunities to utilize viruses for biomedical applications, including tissue engineering, drug/gene delivery, targeting/imaging, etc. [1,2,3,4,5,6]. Virotherapy utilizes engineered viruses via the above technologies as therapeutics to cure diseases [4,7,8]. Oncolytic viruses (OVs) are unique nanomedicines with merits over conventional anti-cancer drugs in terms of their tumor selectivity and killing mechanism [9]. Selectivity can be achieved by either genetic engineering or evolutionary bioselection, which attenuates viral replication in normal cells [10]. Strategies depend on disrupting genes that are critical for the virus to replicate in host cells, but that are limited in normal cells and redundant in cancer cells. For the vaccinia virus (VV), the most popular gene for disruption encodes the *vaccinia thymidine kinase* enzyme (*vTk*); the disruption of this gene results in the preferred replication of VV in cancer cells with high cellular TK expression [11,12].

A refractory cancer one that is resistant to conventional therapy [13,14]; cholangiocarcinoma (CCA) is curable only in its early stage by surgical removal of the tumor mass, but it becomes complex and refractory in later stages, with a dismal prognosis. Therefore, therapeutics with a new mode of action should be developed and employed to treat CCA. OVs have shown promising results for treating various cancers [3,7,15,16]. In our previous study, clinically, JX-594 demonstrated tumor selectivity via viral thymidine kinase (vTK) inactivation because cancer cells have high cellular TK levels due to Epidermal growth factor receptor (EGFR) pathway activation, in which the VV was evolved to replicate selectively in tumors. Engineered VVs have also been shown to successfully kill even cancer stem cell-like cancers using mechanisms different from conventional anti-cancer drugs [15,16]. Specifically, we developed an evolutionary cancer-favoring engineered vaccinia virus (CVV) and demonstrated its ability to selectively and efficiently find and kill stem cell-like colon cancers [15]. CVV was evolved from Wyeth strain vaccinia virus. The evolution was achieved by isolation of Wyeth strain vaccinia virus from the blood of a vaccinia virus-injected VX2 tumor animal model. The isolation of virus was done during the tumor volume reduction, which released viruses in into the serum. From that process, we got evolved virus (called EVV), and subsequently, we engineered EVV by deleting the *Tk* gene to get the virus with enhanced cancer selectivity (called CVV). The higher cancer-favoring characteristics of the CVV were successfully utilized as a strategic therapeutic for metastatic hepatocellular carcinoma (HCC) and induced complete regression of liver tumorigenicity and metastasis to the colon [16]. However, few studies have dealt with CCA in terms of utilizing an OV as a treatment. CCA has a different appearance and tumor localization from other cancers. Furthermore, it is generally thought that cancers have a slightly acidic microenvironment [17,18] compared to normal cells. However, the pH of CCA may be different from general cancers because it is associated with the bile duct, and bile has a slightly alkaline pH (i.e., pH 7–8). 

## 2. Results

### 2.1. Cancer-Favoring, Evolutionarily-Engineered Vaccinia Virus

**Figure 1:**
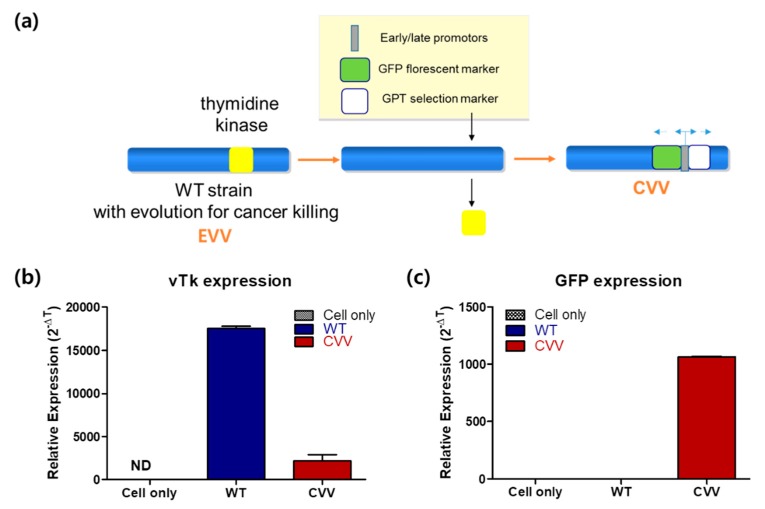
(**a**) The cancer-favoring oncolytic vaccinia virus (CVV) was engineered from the Wyeth strain of the vaccinia virus (VV). The viral gene *vTk* was deleted and the *GFP* and *GPT* selection markers were inserted along the sides of the early/late promoters, and then CVV was evolved and selected after serial culture in serum of infected and responded tumor model. (**b**) vTk expression in WT and CVV-infected HeLa cancer cell lines. Experiments were done in triplicate. (**c**) Confirmation of GFP expression in the CVV-infected HeLa cancer cell line. Experiments were done in duplicate.

The CVV was generated through evolution of the Wyeth strain of the VV, deletion of *thymidine kinase*, and insertion of the *green fluorescence protein* (*GFP*) and *guanine-hypoxanthine phosphoribosyltransferase* (*GPT*) selection markers (Figure 1a). Real-time qPCR results indicated a lower expression of vTK and a higher expression of GFP in CVV-infected cancer cell lines (HeLa). A higher expression of vTK and the lack of expression of GFP were also confirmed in wild-type (WT) VV-infected cell lines (Figure 1b,c). These results confirmed GFP expression in the CVV.

### 2.2. High Replication Efficacy of the CVV in CCA Cell Lines and Tumors 

**Figure 2:**
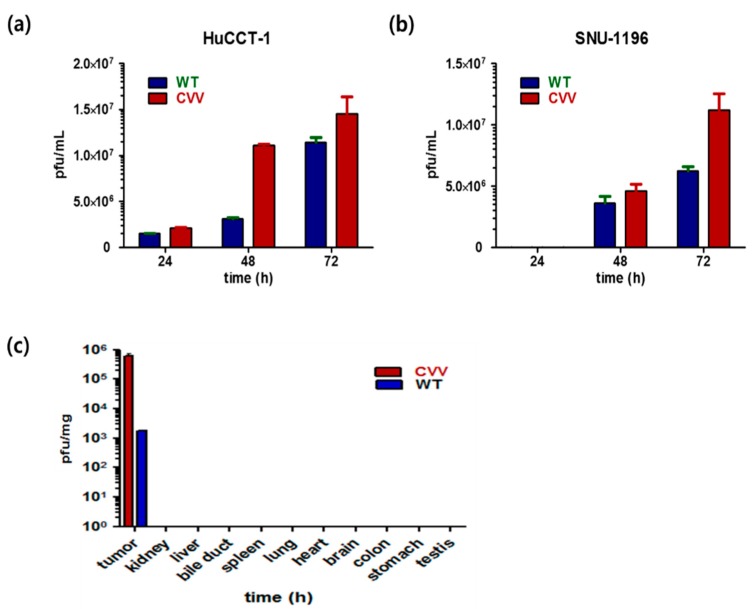
Replication efficacy of the CVV in cholangiocarcinoma (CCA) cell lines and tumors. The WT or CVV (0.1 multiplicity of infection (MOI)) was used to infect HuCCT1 and SNU1196 cells. After 24, 48, and 72 h of infection, cells were harvested. Virus titration was performed using U2OS cells. (**a**) Replication efficacy of the WT and CVV in HuCCT1 cells. Experiments were done in duplicate. (**b**) Replication efficacy of the WT and CVV in SNU1196 cells. Experiments were done in duplicate. (**c**) The bio-distribution of the WT and CVV in HT29-xenograft nude mice confirms the tumor selectivity of the CVV (compared to the WT) in this in vivo model (*n* = 3).

To evaluate the replication efficacy of the CVV in CCA, HuCCT1 and SNU1196 cells were infected with a 0.1 multiplicity of infection (MOI) of the WT or CVV. Infected cell lysates were used to infect U2OS cells for replicated virus titration. The results indicated the superior replication efficacy of the CVV relative to the WT in both cell lines (Figure 2a,b). Furthermore, the results confirmed the robust infectivity of the CVV in the CCA cell lines. The higher efficacy of CVV in cancer can give its greater cancer selectivity. To test in vivo tumor selectivity and safety, tumor tissues and normal organ tissues were harvested 2 weeks after intraperitoneal injection of the virus from HT29-xenografted nude mice. They showed exclusive and robust CVV infection in tumors with negligible effects on the normal bile duct cells (Figure 2c). These results confirmed the tumor-selective infection of the CVV in the CCA model.

### 2.3. Oncolytic Efficacy of the CVV Enhances the Therapeutic Efficacy of Chemotherapeutic Drugs In Vitro 

**Figure 3:**
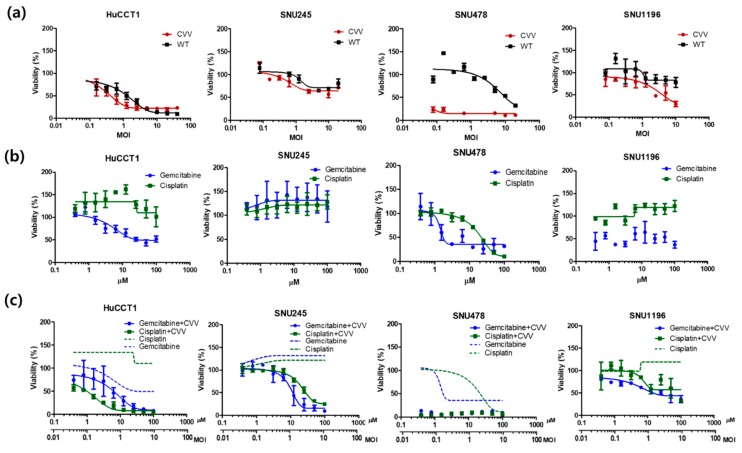
Oncolytic activity of the CVV and its combinations with cisplatin and gemcitabine. (**a**) The viability (%) of four different CCA cell lines against the CVV and WT at different MOIs. (**b**) The viability (%) of four different CCA cell lines against cisplatin and gemcitabine at different concentrations. (**c**) The combination of CVV + cisplatin/CVV + gemcitabine viability (%) in four different CCA cell lines.

Next, to evaluate the cytotoxicity of the CVV in CCA cells, the HuCCT1, SNU245, SNU478, and SNU1196 cell lines were used. Cells were seeded in 96-well plates and the CVV or WT was used for infections at 0.01, 0.1, 1, 10, and 20 MOIs. To monitor cisplatin and gemcitabine toxicity, these drugs were used at concentrations of 0.1–100 µM. The WST-1 assays indicated robust and dose-dependent cytotoxicity of the CVV in four different CCA cell lines (Figure 3a). Gemcitabine showed high cytotoxicity in all cell lines except SNU245 (Figure 3b), and most CCA cells were resistant to cisplatin (Figure 3b). The combined treatment of the CVV and cisplatin showed higher cellular toxicity in HuCCT1 and SNU478 cell lines, while the combined treatment of the CVV and gemcitabine led to higher cellular toxicity in SNU1196 and SNU245 (Figure 3c). These results indicated that CVV monotreatment also works well in CCA, which is very resistant to conventional anticancer drugs, such as gemicitabine and cisplatin. The reason why CVV works well in CCA regardless of the resistance to gemicitabine or cisplatin is because CVV takes cancer selective infection/replication as its cancer killing mechanism, which is different from that of conventional anti-cancer drugs. Therefore, CVV infection in CCA cells may enhance the efficacy of chemotherapeutic agents by overcoming the drug-resistance.

### 2.4. High Expression of Stemness Markers in CCA Cell Lines 

**Figure 4:**
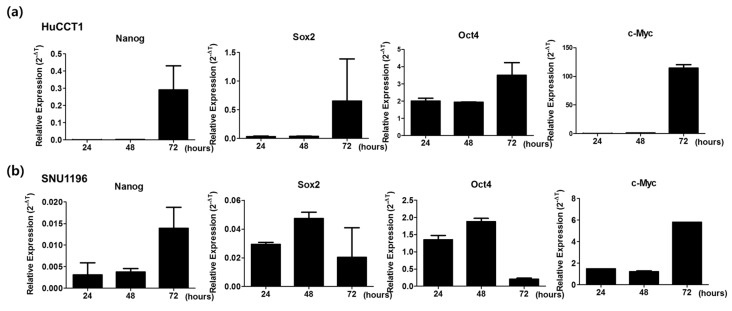
Expression of stemness markers in CCA cell lines. HuCCT1 and SNU1196 cells were cultured in six-well plates. Cells were harvested and total RNA was extracted at three different time intervals: 24, 48, and 72 h. Complementary DNA (cDNA) synthesis was performed from total RNA and used for real-time qPCR expression analysis. (**a**) mRNA expression of stemness markers (Nanog, Sox2, Oct4, and c-Myc) in HuCCT1 cells and (**b**) in SNU1196 cells.

The difficulty of optimal clinical management of CCA is mainly due to its clinical complexity contributed by its multiple cell origins, stemness features, and biliary tumor microenvironment (TME) [19]. To evaluate the stemness of CCA cell lines, mRNA expressions of Nanog, Sox2, Oct4, and c-Myc were analyzed in the HuCCT1 and SNU1196 cell lines. Cells were harvested at three different time intervals (24, 48, and 72 h) for mRNA expression analysis. HuCCT1 cells cultured for 72 h showed the upregulation of all four genes (Figure 4a). Nanog and c-Myc expression were upregulated after 72 h in SNU1196 cells, whereas Sox2 and Oct4 genes were upregulated in cells cultured for 48 h (Figure 4b). mRNA expression of stemness markers confirmed the stemness characteristics of CCA cell lines, which may contribute to drug resistance [15]. 

### 2.5. The Effect of pH on CCA Cell Lines

**Figure 5:**
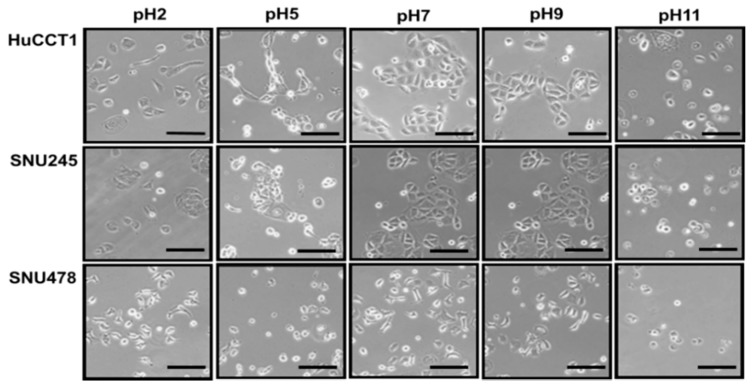
The effect of pH on CCA cells. The pH of the medium was adjusted using HCl and NaOH. The images of cells in different pH media were captured after 24 h under an inverted microscope (scale bar = 100 μm).

To evaluate the effect of pH on CCA cells, the HuCCT1, SNU245, and SNU478 cell lines were used. Cell culture media with the appropriate pH values were used for cell culture. To create an acidic pH, 1 N HCl was used; to create an alkaline pH, 1 N NaOH was used. The cells were stable in media with a pH ranging from 7 to 9 (Figure 5). The survival and spread of CCA cell lines at pH 7–9 indicated the alkaline nature of these cells and their environments.

### 2.6. Alkaline pH Favors CVV Infection in the CCA Cell Line

**Figure 6:**
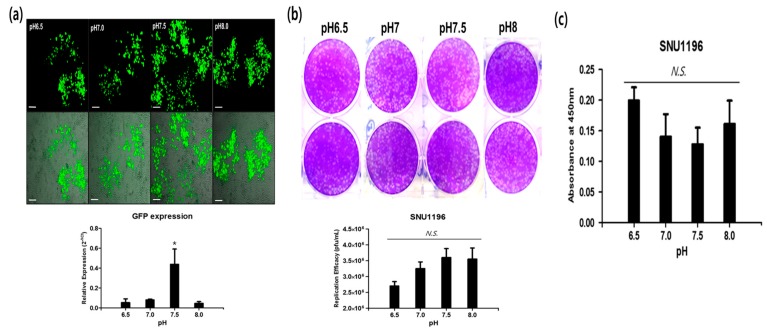
CVV infectivity in CCA. (**a**) Green fluorescence expressions in SNU245 at different pHs (top), 48 h after CVV infection. GFP gene expression from cells cultured in different pHs of media (bottom) (scale bar 200 um); * *p* < 0.05, one-way ANOVA. (**b**) Replication efficacy in SNU1196 cultured in different pHs of media. (**c**) WST-1 assay results of SNU1196 cells cultured in different pHs of media. *N.S.*, * p > 0.05, one-way ANOVA.

To assess the oncolytic efficacy of the CVV under an alkaline pH, HuCCT1 was cultured at four pH conditions ranging from 6.5 to 8.0. The CVV was then infected (0.1 MOI), GFP expression was captured, and cells were harvested for qPCR GFP expression. Green fluorescence expression indicated robust infectivity of the CVV at alkaline pH values of 7.5 and 8.0 (Figure 6a, top). qPCR GFP expression was the highest at pH 7.5 (Figure 6a, bottom). These in vitro results support the robust infectivity of the CVV under an alkaline pH. Replication efficacy in SNU1196 in different pH media (6.5, 7, 7.5, and 8) shows its preference to a slightly alkaline environment (pH 7.5–8) (Figure 6b), which corresponds cell toxicity (decrease of absorbance) at respective pH media (Figure 6c). The differences in replication efficacy and cytotoxicity in SNU1196 were not significant (*p* > 0.05), which is perhaps because CCA is already an alkaline cancer. When we used HeLa (extracellular pH of most cancer is generally considered to be mildly acidic in the rage of 6.4–7.0) [20], the highest replication efficacy of CVV was found in pH 7.5 (* *p* < 0.05, Figure S1). From the results, we propose that the oncolytic activity of the CVV is stable and highly effective at infecting cancer cells in a slightly alkaline microenvironment (pH 7–8), suggesting that CVV is highly suitable for the treatment of CCA. 

### 2.7. Therapeutic Efficacy of CVV in the Xenograft Model 

**Figure 7:**
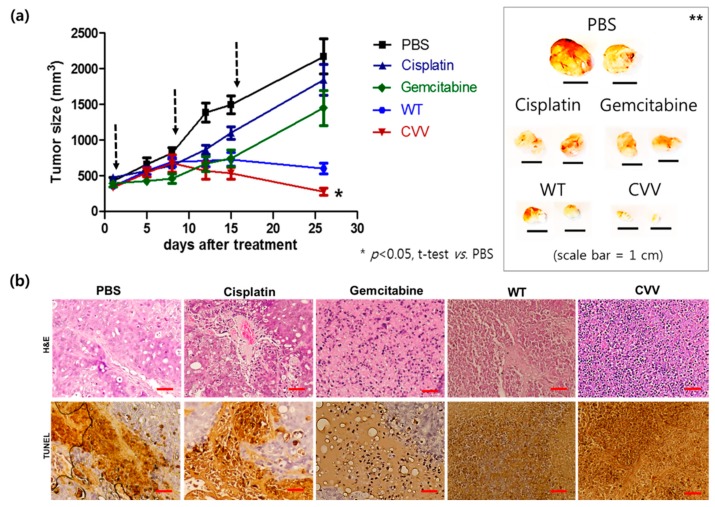
Therapeutic efficacy of the CVV in the SNU1196 xenograft model. (**a**) Tumor size measured in xenograft models treated with PBS, cisplatin, gemcitabine, and WT or CVV. SNU1196 tumors were induced in nude mice. When the tumor volume reached 300 mm^3^, treatment was started. The treatment scheduled days are noted with downwards arrows. Tumor volume in nude mice after treatment (left). Tumor volume after treatment (left). Image of SNU1196 tumors 4 weeks after treatment (right). One-paired t-test were used to detect the significance in tumor volume growth (* *p* < 0.05) and one-way ANOVA test shows that tumor volume at day 27 are significantly different between groups (** *p* <0.0001). (**b**) Hematoxylin and eosin (H&E) staining and transferase dUTP nick end labeling (TUNEL) results for each tumor tissue of the xenograft mice treated with PBS, cisplatin, gemcitabine, and WT or CVV. (scale bar 100 um)

To prove the oncolytic efficacy of the CVV in CCA in vivo, the SNU1196 xenograft model was used. SNU1196 cells (2 × 10^7^) were injected subcutaneously into the left flank of nude mice. Mice were categorized into five groups (PBS, cisplatin, gemcitabine, WT, and CVV), with five mice per group and were treated as per Section 2.6. After the tumor volume reached 300 mm^3^, 100 μL of PBS, 1 × 10^6^ plaque-forming units of the CVV or WT, and 25 mg/kg of cisplatin or gemcitabine were injected intratumorally to the corresponding mice groups once per week. The tumor volume (mm^3^) was measured twice per week using the formula: L × W2/2, where L is the tumor length and W is the tumor width. A slower tumor burden was observed in CVV-treated mice than in PBS-treated mice (*p* < 0.05, Figure 7a). Gemcitabine may work more rapidly (approximately up to 10 days after treatment), but surviving, drug-resistant stem cells like CCA started to grow after that. In addition, regression of tumor burden was observed only in the viral (WT and CVV) treatment group. Additionally, total cancer cell lysis was easily found within the tumor mass only in the viral treatment groups. As expected, the highest efficacy was found in the CVV group (tumor size: PBS > cisplatin > gemcitabin > WT > CVV group). Hematoxylin and eosin (H&E) staining results of tumor mass and terminal deoxynucleotidyl transferase dUTP nick end labeling (TUNEL) staining results of tumor mass of each group indicated that CVV most effectively induced apoptosis in the tumor mass (Figure 7b). This result confirmed the therapeutic efficacy of the CVV in the CCA xenograft model.

## 3. Discussion

Patients with CCA who undergo chemotherapy show only a partial response rate and have a low median survival [13]. Most of these therapies target various growth factors and their signaling cascades [21]. However, the induction of systemic antitumor activity is questionable in these approaches. Oncolytic virotherapy uses live recombinant or natural viruses to selectively kill cancer cells. In addition, OVs can induce both innate and adoptive immunity against cancer cells [9,22]. Some studies have shown the therapeutic efficacy and diagnostic ability of OVs against CCA [23,24,25]. However, further clinical applications for CCA are needed. Here, we considered the alkaline nature of CCA tumors in the intrahepatic bile duct, together with the cancer-favoring characteristics of CVV overcoming drug-resistance issues. Our previous studies proved the therapeutic efficacy of the CVV against stem cell-like colon cancer and metastatic hepatocellular carcinoma (HCC) due to its distinct cancer-killing pathway and cancer-favoring characteristics [15,16]. Upregulated GFP and downregulated vTk expression from CVV-infected cell lines confirmed the infectivity of the CVV (Figure 1b,c). Furthermore, replication efficacy assays using two different CCA cell lines demonstrated the robust replication efficacy of the CVV (Figure 2a,b) in CCAs, as shown by exclusive infection of the CVV in tumors in the HT29-xenograft mice model (Figure 2c). These results suggest the selective oncolytic activity of the CVV on CCA tumors, while sparing normal cells. Cell viability assays using different CCA cell lines indicated robust oncolytic activity and cytotoxicity in combination with cisplatin or gemcitabine (Figure 3a–c). The therapeutic efficacy of the CVV against stem cell-like colon cancer was proven in a previous study [15]. To prove the therapeutic efficacy of the CVV against stem cell-like CCA tumors, upregulated mRNA expressions of stemness markers, such as Nanog, Sox2, Oct4, and c-Myc, were shown in the HuCCT1 and SNU1196 cell lines (Figure 4a); hence, we used SNU1196 to evaluate the therapeutic efficacy of the CVV in the xenograft model. We considered the alkaline environment of CCA tumors in the bile duct to check whether extracellular pH modulation, in addition to enhanced cancer selectivity of CVV, would be able to overcome the drug-resistance and complexity of the TME, would enhance the therapeutic activity of CVV on these refractory cancers. Three different CCA cell lines were cultured in media with four different pH values, and the normal proliferation of HuCCT1 cells in a slightly alkaline pH environment was observed (Figure 5). GFP expression of the CVV in terms of green fluorescence and mRNA expression in HuCCT1 cells confirmed the replication and oncolytic efficacy of the CVV in SNU1196 were highest at pH 7.5 (Figure 6). These results support that the oncolytic efficacy of the CVV on CCA cell lines is enhanced with the alkaline pH in vitro. In the xenograft model, the slower growth and total lysis (apoptosis) within SNU1196 tumor mass in CVV-treated nude mice indicated the therapeutic efficacy of the CVV against CCA tumors (Figure 7). 

It is widely accepted that the extracellular pH (pH_e_) of cancer cells is more acidic than normal cells and the intracellular pH (pH_i_) of them is neutral or even more alkaline than that in normal. Recently, some suggested that an approach to manipulating pH_e_ could be achieved by the targeted delivery of alkaline nanoparticles to the tumor tissue as a future clinical application [26]. As an aspect of the impact of TME and stem-like plasticity in CCA, this pH_e_ modulation approach in conjunction with CVV is likely to be very effective in cancer therapy. This application is very applicable for other cancer cell types as well. The promise of this therapeutic potential will be confirmed with a clinical assessment of its efficacy and safety in further studies. Before the clinical evaluation, therapeutic efficacy tests of CVV in syngeneic and orthotopic animal models should be considered. In clinical studies, administration of the virus, how to modulate pH_e_ in surrounding cancer tissue, and optimization in corresponding cancer types should be also considered.

## 4. Materials and Methods 

### 4.1. Cell Culture and Virus

Human CCA cell lines (HuCCT1, SNU245, SNU478, and SNU1196) were obtained from a Korean cell line bank (KCLRF, Seoul, South Korea). All cell culture media and their related reagents were obtained from Welgene (Gyeongsan, South Korea). These cells were thawed and cultured in Roswell Park Memorial Institute (RPMI) 1640 medium (10% fetal bovine serum (FBS) and 1% penicillin/streptomycin(P/S)) in the presence of 5% CO_2_ at 37 °C in an incubator. For HeLa and HT29 cells, they were thawed and cultured in Dulbecco Modified Eagle medium (DMEM) (10% FBS and 1% P/S). Cells were subcultured when they achieved 70% confluence. The CVV was engineered as per Figure 1a. Phase-contrast images of the cells were taken by a microscope (EVOS Cell Imaging Systems, Thermo Fisher Scientific, Waltham, MA, USA).

### 4.2. Replication Efficacy Assay

To assess the replication efficacy of the CVV in CCA (HuCCT1 and SNU1196), 2 × 10^5^ cells per well were seeded in six-well plates. After 90% confluence, 0.1 MOI of the CVV was used to infect cells. Cells were harvested after 72 h and frozen at −80 °C for virus titration. For virus titration, 2 × 10^5^ U2OS (ATCC® HTB-96™, Manassas, VA, USA) cells per well were seeded in six-well plates. Harvested CVV-infected cell lysate was used to infect U2OS cells by serial dilution. At 72 h post infection, cells were stained with 0.1% crystal violet solution. After 2–4 h, destaining was performed and the plates were dried to count the plaques. 

### 4.3. WST-1 Assay

The WST-1 assay was used to evaluate the cellular toxicity of the CVV in various CCA cell lines. First, 1 × 10^4^ cells per well were seeded in 96-well plates. After 24 h, chemotherapeutic drugs or the CVV were used to infect cells at different concentrations (0.1, 1.0, 10, 100, and 1000 µM, and 0.01, 0.1, 1.0, 10, and 100 MOI, respectively) in serum-free media. Serum-free media were replaced with normal media after 2 h. At 72 h post infection, cells were analyzed using the WST-1 assay (EzCytotox, ITSBIO, Seoul, Korea). As per the manufacturer’s instructions, absorbance was measured at 450 and 680 nm (reference wavelengths).

### 4.4. Cells Culturing in pH-Adjusted Media 

To adjust pH of the media, 1N NaoH or 1 N HCl was used. HuCCT1, SNU245, SNU245, and SNU1196 cells were seeded in six-well plates. After 24 h of seeding, the normal media (RPMI1640, 10%FBS, 1%P/S) was replaced with corresponding pH-adjusted media (RPMI1640; no FBS; pH: 2.0, 5.0, 6.0, 6.5, 7.0, 7.5, 8.0, 9.0, and 11.0). Cells were kept in 2 h in pH-adjusted media. After this, pH-adjusted media was replaced with normal media. The proliferation of cells was noted after 24 h using inverted microscope.

### 4.5. Real-Time PCR Assay

Total RNA was extracted from cell lines using Trizol reagent (Thermo Fisher Scientific Korea, Seoul, South Korea). To confirm RNA purity, absorbance was measured at 260 and 280 nm. For cDNA synthesis, 2 μg total RNA was used with the PrimeScript 1st Strand cDNA Synthesis Kit (Takara Korea, Seoul, Korea). For the real-time polymerase chain reaction (PCR), 1 μg cDNA was used with the SYBR-Green quantitative PCR (qPCR) mixture (Roche, Basel, Switzerland) reagent. A LightCycler 96 Real-Time PCR System (Roche) was used to perform the real-time PCR assay. The program consisted of a 40-cycle amplification at 95 °C for 20 s, 60 °C for 20 s, and 72 °C for 25 s. β-actin mRNA expression was used to normalize the expression of the selected genes using the 2^−ΔΔCt^ method.

### 4.6. Animal Study

All animal experiments were approved by the Institutional Animal Care and Use Committee of Pusan National University (PNU-2017-1533). Nude mice were purchased from Orient (Gapyeong, South Korea). SNU1196 (2 × 10^7^ cells/100 μL) was injected subcutaneously into the left flank of nude mice. Mice were divided into five groups; namely, PBS, cisplatin, gemcitabine, WT, and CVV, with five mice per group. The tumor volume (mm^3^) was measured twice per week using the formula: L × W2/2, where L is the tumor length and W is the tumor width. After the tumor volume reached 300 mm^3^, 100 μL of PBS, 1 × 10^6^ plaque-forming units of the CVV or WT, cisplatin (25 mg/kg, Selleckchem, Houston, TX, USA), and gemcitabine (Merck, Darmstadt, Germany) were injected intratumorally to the corresponding mice groups once per week. We used 10^6^ plaque-forming units (pfu) virus/mouse because CVVs have a high replication rate [15,16] and the infectious dose of the WT or JX594 viruses used in a previous in vivo study was more than 10^7^ pfu [27]. After four weeks of injection, mice were euthanized and the tumors were harvested for imaging. For the bio-distribution of viruses, organs and tumors were collected from HT29-xenograft nude mice, which received 1 × 10^6^ plaque-forming units of the CVV or WT intraperitoneally and were sacrificed after 2 weeks.

### 4.7. H&E staining and TUNEL Assay 

To assay tomor generation and morphological characterics, H&E staining and TUNEL assays were performed on paraffin-embedded tumor sections. Images were acquired using EVOS Cell Imaging Systems (Thermo Fisher Scientific). 

### 4.8. Statistical Analysis 

Student’s unpaired *t*-tests were used to analyze the statistical significance of the SNU1196 tumor volumes in nude mice. The level of statistical significance was set at *p* < 0.05. 

## 5. Conclusions

In conclusion, the CCA cell lines and xenograft-based results demonstrated the oncolytic efficacy of the CVV and confirmed its therapeutic efficacy in different circumstances in relation to the stem cell-like characteristics and the alkaline nature of CCA tumors. The findings of this study support the fact that CVV-based virotherapy is also suitable for the treatment of drug-resistant CCA.

## Supplementary Materials

The following are available online at https://www.mdpi.com/2072-6694/11/11/1667/s1, Figure S1: Replication efficacy in HeLa cultured in different pHs of media. * *p* < 0.05, one-way ANOVA.
