# Supplementary material for: A Cancer-Favoring, Engineered Vaccinia Virus for Cholangiocarcinoma
Source: Cancers (Basel). 2019 Oct 27;11(11):1667. doi: 10.3390/cancers11111667 (PMC6896061; doi:10.3390/cancers11111667)
Supplement: Supplementary file 1 [file cancers-11-01667-s001.pdf]

Supplementary Materials

A Cancer-Favoring Engineered Vaccinia Virus for Cholangiocarcinoma

So Young Yoo, Narayanasamy Badrinath, Hye Lim Lee, Jeong Heo and Dae-Hwan Kang

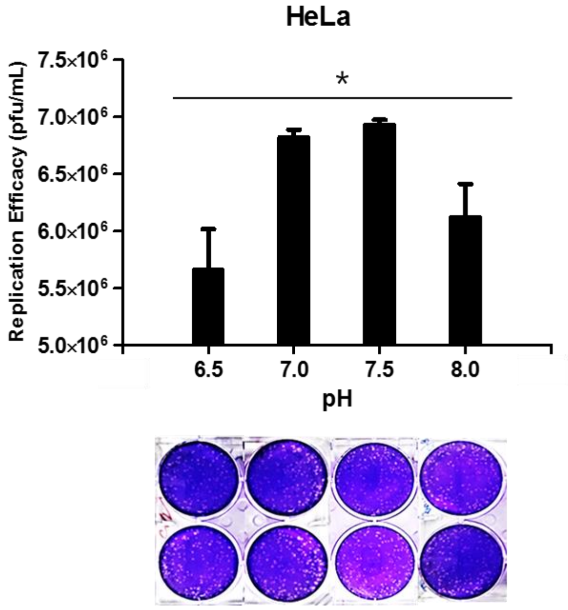

Figure 1. Replication efficacy in HeLa cultured in different pHs of media. $^{*}p < 0.05$, one-way ANOVA.

© 2019 by the authors. Submitted for possible open access publication under the terms and conditions of the Creative Commons Attribution (CC BY) license (http://creativecommons.org/licenses/by/4.0/).
